# A Novel Role for Aquaporin-5 in Enhancing Microtubule Organization and Stability

**DOI:** 10.1371/journal.pone.0038717

**Published:** 2012-06-08

**Authors:** Venkataramana K. Sidhaye, Eric Chau, Vasudha Srivastava, Srinivas Sirimalle, Chinmayee Balabhadrapatruni, Neil R. Aggarwal, Franco R. D'Alessio, Douglas N. Robinson, Landon S. King

**Affiliations:** 1 Department of Medicine, Division of Pulmonary and Critical Care Medicine, The Johns Hopkins University School of Medicine, Johns Hopkins University, Baltimore, Maryland, United States of America; 2 Department of Cell Biology, The Johns Hopkins University School of Medicine, Johns Hopkins University, Baltimore, Maryland, United States of America; 3 Department of Biological Chemistry, The Johns Hopkins University School of Medicine, Johns Hopkins University, Baltimore, Maryland, United States of America; University of Pittsburgh, School of Medicine, United States of America

## Abstract

Aquaporin-5 (AQP5) is a water-specific channel located on the apical surface of airway epithelial cells. In addition to regulating transcellular water permeability, AQP5 can regulate paracellular permeability, though the mechanisms by which this occurs have not been determined. Microtubules also regulate paracellular permeability. Here, we report that AQP5 promotes microtubule assembly and helps maintain the assembled microtubule steady state levels with slower turnover dynamics *in cells*. Specifically, reduced levels of AQP5 correlated with lower levels of assembled microtubules and decreased paracellular permeability. In contrast, overexpression of AQP5 increased assembly of microtubules, with evidence of increased MT stability, and promoted the formation of long straight microtubules in the apical domain of the epithelial cells. These findings indicate that AQP5-mediated regulation of microtubule dynamics modulates airway epithelial barrier properties and epithelial function.

## Introduction

Aquaporin-5 (AQP5) is a water-specific channel located on the apical membrane of epithelial cells in several sites in mammals, including corneal and pancreatic epithelium, secretory cells in salivary and lacrimal glands, and airway submucosal glands, bronchial epithelium and type I pneumocytes of the respiratory tract. The primary focus of studies on AQP5 function has been related to its water selectivity and its role in conveying a high degree of membrane water permeability [1], [2], [3], [4], although other roles have also been suggested, including regulation of paracellular permeability [5], [6], cell proliferation [7], [8], or cell migration [8], [9], [10]. While regulation of local water flux could contribute to these processes, the specific mechanisms by which these processes are mediated are not defined.

Airway epithelial cells can dynamically regulate paracellular permeability in response to both physiologic and pathologic stimuli [6], [11]. AQP5 can regulate paracellular permeability in primary airway epithelial cells [6]. Regulation of paracellular permeability has two main functions. First, it serves to gate the passage of ions and macromolecules through the paracellular pathway, and restricts access of these molecules to subepithelial tissues. In addition, it separates and regulates access between the apical and basolateral membrane domains of polarized epithelia. [12], [13]. Dynamic changes in airway epithelial paracellular permeability allow for receptor-ligand access across the airway epithelium, and thereby regulate cell signaling [11]. In airway epithelial cells, altered AQP5 abundance is associated with changes in actin organization and in desmoplakin localization, which could contribute to the changes in paracellular permeability. [6]. Others have shown a similar change in paracellular permeability regulated by AQP5 in salivary glands, however in that system it was associated with alterations in claudin-7, claudin-3, and occludin [5]. In both studies, mechanisms by which AQP5 altered these proteins were not further elucidated. In polarized epithelium, adhesion between adjacent cells is mediated by intercellular junctions, namely, tight junctions, adherens junctions, and desmosomes [14]. These structures are composed of adhesive and scaffolding proteins that are anchored to different cytoskeletal structures such as actin filaments, intermediate filaments, and microtubules (MTs). In response to many stimuli, changes in epithelial permeability result from cytoskeletal rearrangement that modifies these intercellular junctions [15]. However, the effects of cytoskeletal rearrangements on barrier function may be cell type specific. Airway epithelial cells have decreases in paracellular permeability when exposed to MT depolymerizing agents such as nocodazole [16]. It has been shown that MT dynamics regulate actin organization and adherens junctions in epithelial cells [16], [17], thereby contributing to changes in paracellular permeability. In direct contrast, microtubule disassembly has been associated with increases in paracellular permeability in the endothelium [18], [19], [20]. Mechanisms mediating these differing responses are unclear, but there is precedence for differential epithelial and endothelial barrier responses to stimuli such as thrombin [21].

Using both *in vivo* and *in vitro* models, we have demonstrated that low levels of shear stress, as sensed by the airway epithelium during quiet, or tidal volume breathing, decreased paracellular permeability. Shear stress decreased AQP5 and paracellular permeability, while overexpression of AQP5 increased paracellular permeability [6]. Lorenowicz and colleagues described that microtubule depolymerization results in decreased paracellular permeability, so we sought to determine if shear-induced changes in permeability were in part mediated microtubule (MT) dynamics [16].

In this study, we identify a novel role for AQP5 in directly regulating MT polymerization by increasing MT stability, an effect that appears to be specific to AQP5 as AQP1 does not show these properties. The AQP5 carboxyl-terminus is sufficient to promote microtubule assembly, suggesting that the effect is not dependent on water transport properties.

## Results

### Shear stress increases microtubule depolymerization and decreases paracellular permeability in NHBE cells

**Figure 1:**
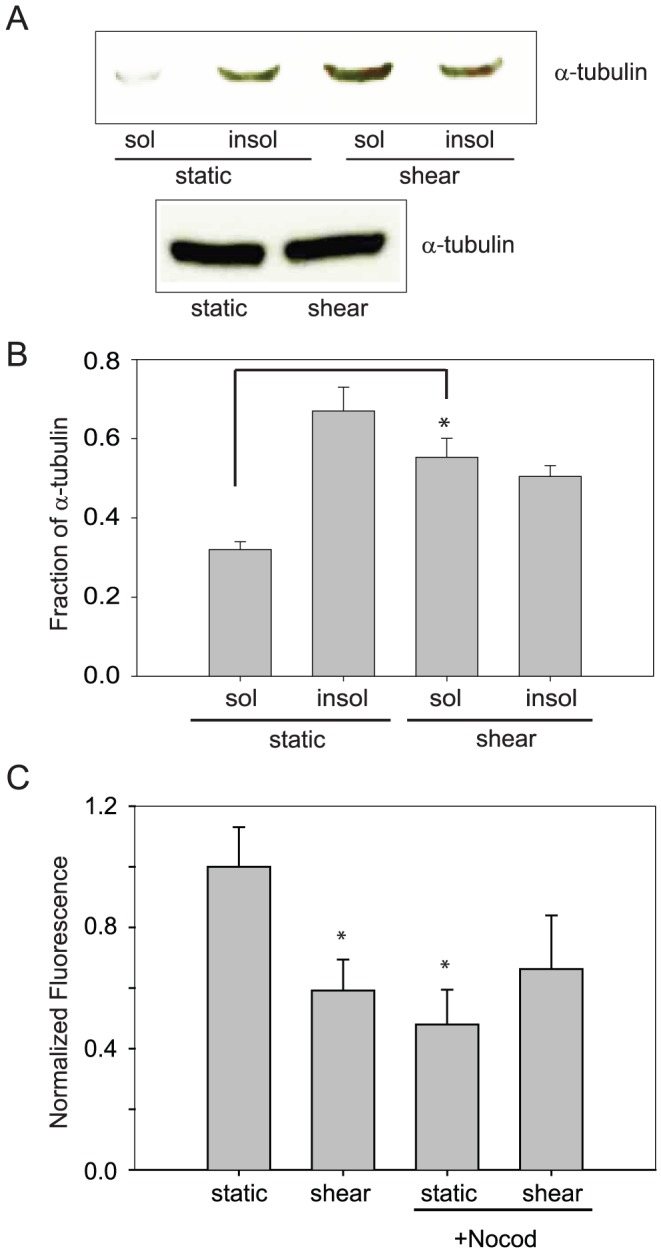
Shear stress and MT depolymerization are associated with decreased airway epithelial paracellular permeability and shear stress results in increased MT depolymerization. A. Exposure to shear stress caused an increase in depolymerized, or soluble tubulin as assessed by western blotting, without a change in total tubulin. B. From densitometry analysis, the level of soluble tubulin was increased significantly in cells exposed to shear stress as compared to static cells. (n=6; * *p*<0.025 with ANOVA one-way) C. NHBE cells exposed to nocodazole (20 µM) had a decrease in FITC-dextran permeability, similar to that seen after exposure to shear stress. After treatment with nocodazole, exposure to shear stress led to no further decrease in paracellular permeability (n=5; * *p*<0.025 with ANOVA one-way)

To identify a role for MT levels in the shear-induced decreased paracellular permeability, we used well established biochemical techniques whereby detergent soluble, disassembled tubulin is extracted from permeabilized cells [22], [23], [24], [25]. Assembled MT fractions were then quantified by immunoblotting, and equal loading and transfer was confirmed with Ponceau S staining of the membrane (not shown). We measured soluble and insoluble fractions of tubulin from NHBE cells after exposure to either static or shear conditions (Fig. 1A, B). Shear stress increased the soluble tubulin fraction as compared to static cells while total tubulin levels remained unchanged (Fig. 1A). Others have suggested that a reduction in assembled MTs leads to decreased paracellular permeability in airway epithelial cells, as measured by transepithelial resistance [16]. Following exposure of NHBE cells to nocodazole (a MT depolymerizing agent) for 1 h, FITC-dextran permeability decreased, similar to that seen after exposure to shear stress (Fig. 1C). After treatment of NHBE cells with nocodazole, shear stress produced no further decrease in paracellular permeability.

### AQP5 alters microtubule stability

**Figure 2:**
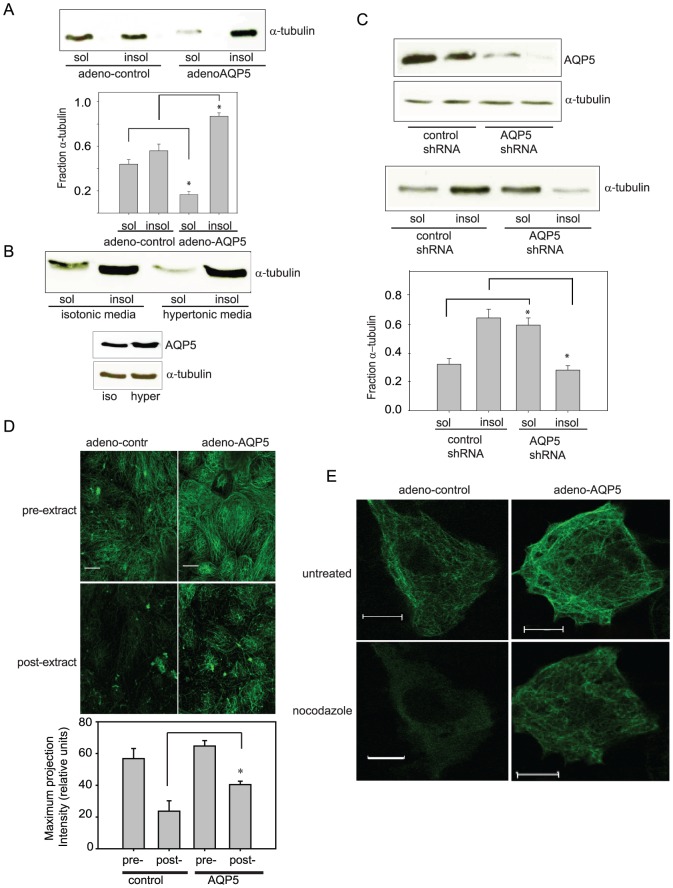
Molecular manipulation of AQP5 altered the MT soluble fraction. A. Transduction of adeno-AQP5 in 16HBE cells led to a significant decrease in soluble tubulin fraction. A representative immunoblot is shown. A bar graph shows the quantification by densitometry (n=4; * *p*<0.025 with ANOVA one-way). B. In NHBE cells, treatment with hypertonic media led to an increase in the insoluble MT fraction, compared to isotonic control. Hypertonic media caused an increase in total AQP5. (n=2) C. In NHBE cells, knockdown of AQP5 by adenoviral transduction led to an increase in the soluble MT fraction, compared to a scrambled control. Representative immunoblots and bar graphs are shown. (n=4; * *p*<0.025 with ANOVA one-way). D. Immunofluorescence of tubulin was performed on HBE cells transduced with either control adenovirus or adeno-AQP5 pre- or post- soluble tubulin extraction without a change in intensity settings. In cells with transduced with adeno-AQP5, there is significantly more microtubules visualized post-extraction. Quantitative analysis of these images were performed by obtaining the whole field intensity of the maximum projection intensity of the fields (n=10, *p<0.01) E. Overexpression of AQP5 caused endogenous MTs to be more resistant to nocodazole treatment. 16HBE cells were transduced with either control adenovirus or adeno-AQP5 and MT expression was compared before and after nocodazole treatment (10 µM). No clear change in tubulin polymerization in adeno- control or adeno-AQP5 expressing cells was observed. After 10 min of treatment with nocodazole, a considerable decrease in polymerized tubulin was observed in adeno-control expressing cells. However, the adeno-AQP5 infected cells maintained a substantial level of polymerized tubulin. Scale bar, 10 µm; applies to each frame. (n=10, per condition. Representative figure shown).

We identified that shear-mediated reduction in paracellular permeability resulted from decreased AQP5 abundance, while overexpression of AQP5 in 16HBE cells (which do not express endogenous AQP5) increases paracellular permeability [6]. To determine whether changes in AQP5 abundance alter assembled MT steady state levels, we transduced either control adenovirus or adeno-AQP5 into 16HBE cells, and assessed soluble and insoluble tubulin fractions. The efficiency of adenoviral transduction of 16HBE cells was almost 100%, as anticipated [6]. Transduction of adeno-AQP5 decreased the soluble tubulin fraction, shown by densitometry analysis (Fig. 2A). In order to induce increased AQP5, we treated NHBE cells with hypertonic media [26]. Hypertonic exposure lead to an increase in total AQP5 as well as an increase in the insoluble fraction of tubulin (Fig. 2B) To confirm that decreased AQP5 result in decreased assembled MT levels, we knocked down AQP5 in NHBE cells using a lentiviral shRNA transduction. We achieved 80–90% knockdown (Fig. 2B) and significantly increased the soluble tubulin fraction (Fig. 2C). We visualized the impact of AQP5 on MT stability by expressing AQP5 in 16HBE cells using adenoviruses. AQP5 expression increased levels of stable, assembled MTs, which were visualized by immunofluorescence after cell extraction (Fig. 2D). To further assess the effects of AQP5 on tubulin stability, we exposed adeno-control or adeno-AQP5-transduced 16HBE cells to nocodazole before visualizing the MTs by immunofluorescence. After 10 min of nocodazole treatment, the assembled MTs decreased in control cells. However, adeno-AQP5 cells maintained more assembled MT arrays after nocodazole treatment compared to adeno-control cells (Fig. 2E).

**Figure 3:**
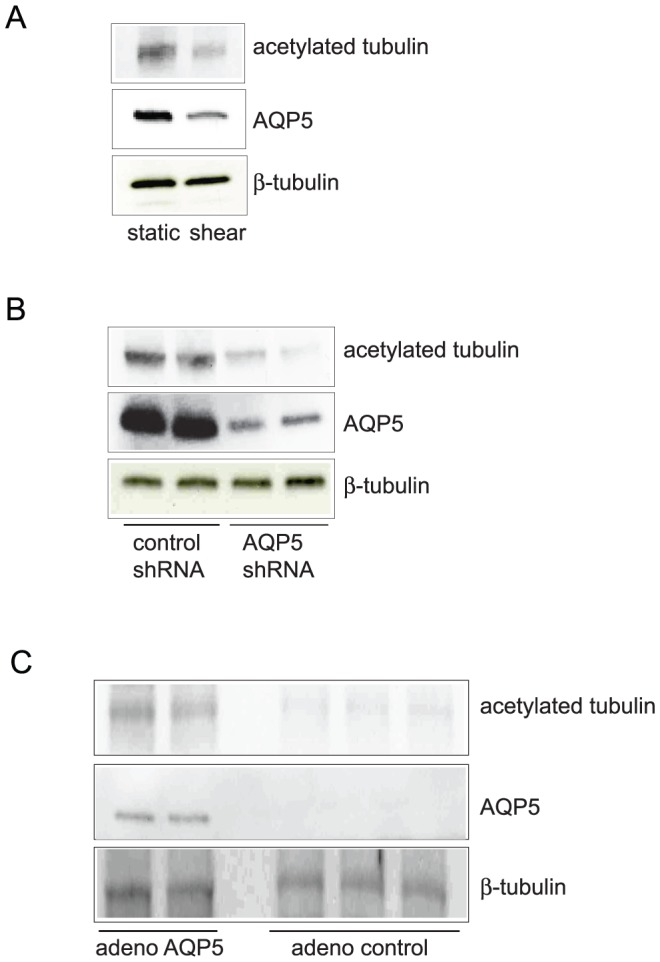
Changes in AQP5 are associated with altered acetylated tubulin (n=6, per condition, representative blots shown). A. Shear stress, which causes decreased AQP5, led to decreased acetylated tubulin with no change in total tubulin. B. In NHBE cells, AQP5 knockdown resulted in decreased acetylated tubulin, with no change in total tubulin. C. Overexpression of AQP5 resulted in increased acetylated tubulin in 16HBE cells.

Increased tubulin acetylation is associated with increased tubulin stability [27], [28], [29], [30]. To further confirm that AQP5 expression increased tubulin stability, we assessed tubulin acetylation in response to AQP5 manipulation. NHBE cells were exposed to static and shear conditions. Physiologic shear stress, which decreased AQP5 abundance, also caused a decrease in acetylated tubulin (Fig. 3A). AQP5 knockdown in NHBE cells, similarly resulted in decreased tubulin acetylation (Fig. 3B). Finally, overexpression of AQP5 in 16HBE cells resulted in increased tubulin acetylation (Fig. 3C).

### AQP5 directly interacts with tubulin to promote assembly and stabilize MTs

**Figure 4:**
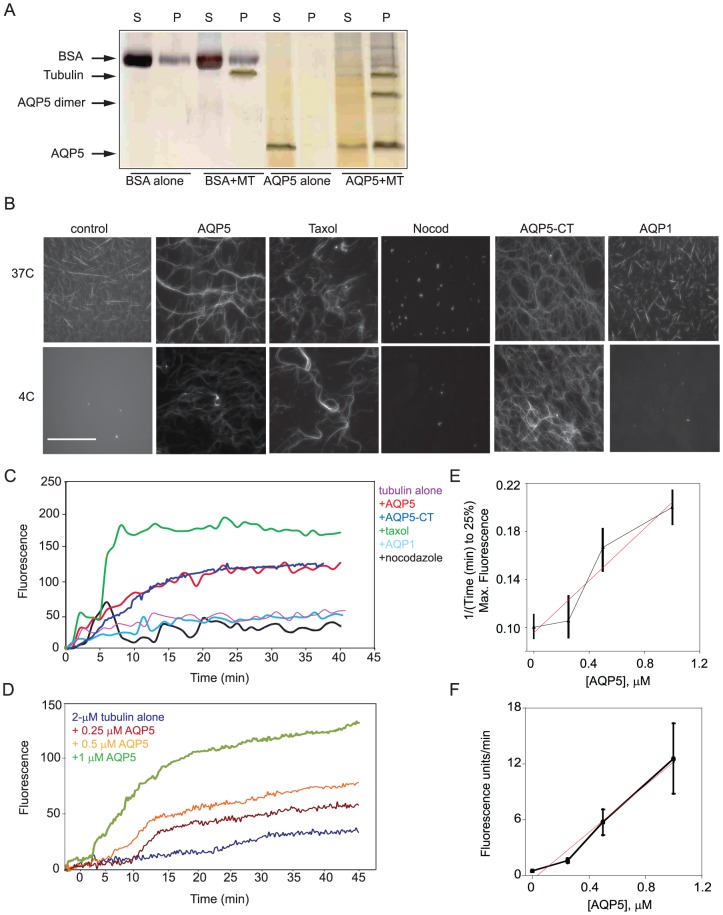
AQP5 purified protein both associated with MTs and altered MT polymerization (all experiments have a minimum of 4 replicates). A. Using a spin down co-sedimentation assay, polymerized tubulin was primarily in the pellet. AQP5, in the absence of MTs remains in the supernatant, however, after the addition of MTs it was primarily in the pellet. In contrast, as a negative control, BSA was primarily in the supernatant and addition of MTs does not alter its localization. B. Using fluorescence microscopy, changes in MT assembly were visibly apparent. At 37 C, MTs assembled to different extents under all conditions, except in the presence of nocodazole. Upon shifting the temperature to 4 C, MTs disassembled in the tubulin-alone control and in the presence of AQP1. However, MTs remained assembled in the presence of AQP5, taxol and AQP5-CT. (scale bar 100 µM) C. In a fluorescence-based polymerization assay, AQP5 (1 µM) and AQP5-CT, like taxol, increased MT polymerization whereas AQP1 (1 µM) failed to support MT assembly as compared to tubulin alone (2 µM). Nocodazole also inhibited assembly. Taxol (30 µM) and nocodazole (30 µM) were added as positive and negative controls, respectively. D. Addition of increasing concentrations of AQP5 increased the rate and extent of MT assembly in the fluorescence assay. E. Analysis of the fluorescence assembly assay data revealed that the inverse time to 25% assembly, which is largely dominated by nucleation, increased with increasing AQP5 concentration. The slope was 0.35 µM^−1^ min^−1^. F. Analysis of the rapid, linear growth phase showed an increasing rate of polymer assembly as a function of AQP5 concentration. The slope was 13 units µM^−1^ min^−1^.

Our cell culture studies suggested that overexpression of AQP5 stabilizes MTs. To determine if AQP5 mediated MT stabilization by direct interactions between AQP5 and tubulin, we examined the effect of purified human AQP5 on tubulin polymerization *in vitro*. We first used bulk co-sedimentation assays where polymerized tubulin sediments into the pellet fraction. Without polymerized tubulin, AQP5 remained in the soluble (supernatant) fraction. However, upon addition of MTs, AQP5 separated into the pellet fraction. Bovine serum albumin (BSA; negative control) remained primarily in the supernatant fraction with and without MTs (Fig. 4A). These results indicate that purified AQP5 can associate with MTs. We note that in the pellet fractions, we also observed a protein species in the AQP5+MT samples which by westerns is consistent with being an AQP5 dimer, suggesting that AQP5 dimers may associate with MTs.

Using fluorescence microscopy, we tested if the addition of purified AQP5 could promote microtubule stability (Fig. 4B). At 37°C, we observed assembled MTs under all conditions except with nocodazole. However, upon shift to 4°C where MTs normally fail to assemble due to their cold sensitivity, AQP5, like taxol, maintained assembled MTs. We found that the carboxyl-terminal domain of AQP5 (final 40 a.a.; AQP5-CT) was sufficient to maintain MT stability.

Addition of either AQP5 or the AQP5-CT to soluble tubulin promoted tubulin polymerization (Fig. 4C). In contrast, the addition of AQP1 neither increased polymerization at 37°C (Fig. 3C) nor stabilized MTs at 4°C (Fig. 4B). Nocodazole was used as a control in both assays, demonstrating that the fluorescence signal reflected MT assembly (Fig. 4B, C). The rate and level of MT assembly increased with increasing AQP5 concentration (0.25, 0.5 and 1 µM) (Fig. 4D). The available concentrations of purified AQP5 limited our ability to evaluate higher concentrations and ratios. Analysis of the fluorescence assembly assay data revealed that the inverse time to 25% assembly, which is largely dominated by nucleation, increased with increasing AQP5 concentrations with a slope of 0.35 µM^−1^ min^−1^ (Fig. 4E), suggesting that purified AQP5 alters MT nucleation in the *in vitro* assay. Increasing AQP5 concentrations had a stronger effect on the MT growth (elongation) phase though this regime did not readily fit a single exponential. Instead, the rapid phase was better described as linear with a slope of 13 units·µM^−1^ min^−1^ (Fig. 4F). Thus, AQP5 has a strong effect on the growth and stability of MTs with a more subtle, but detectable, effect on MT nucleation.

### AQP5 promotes microtubule stability in cells

**Figure 5:**
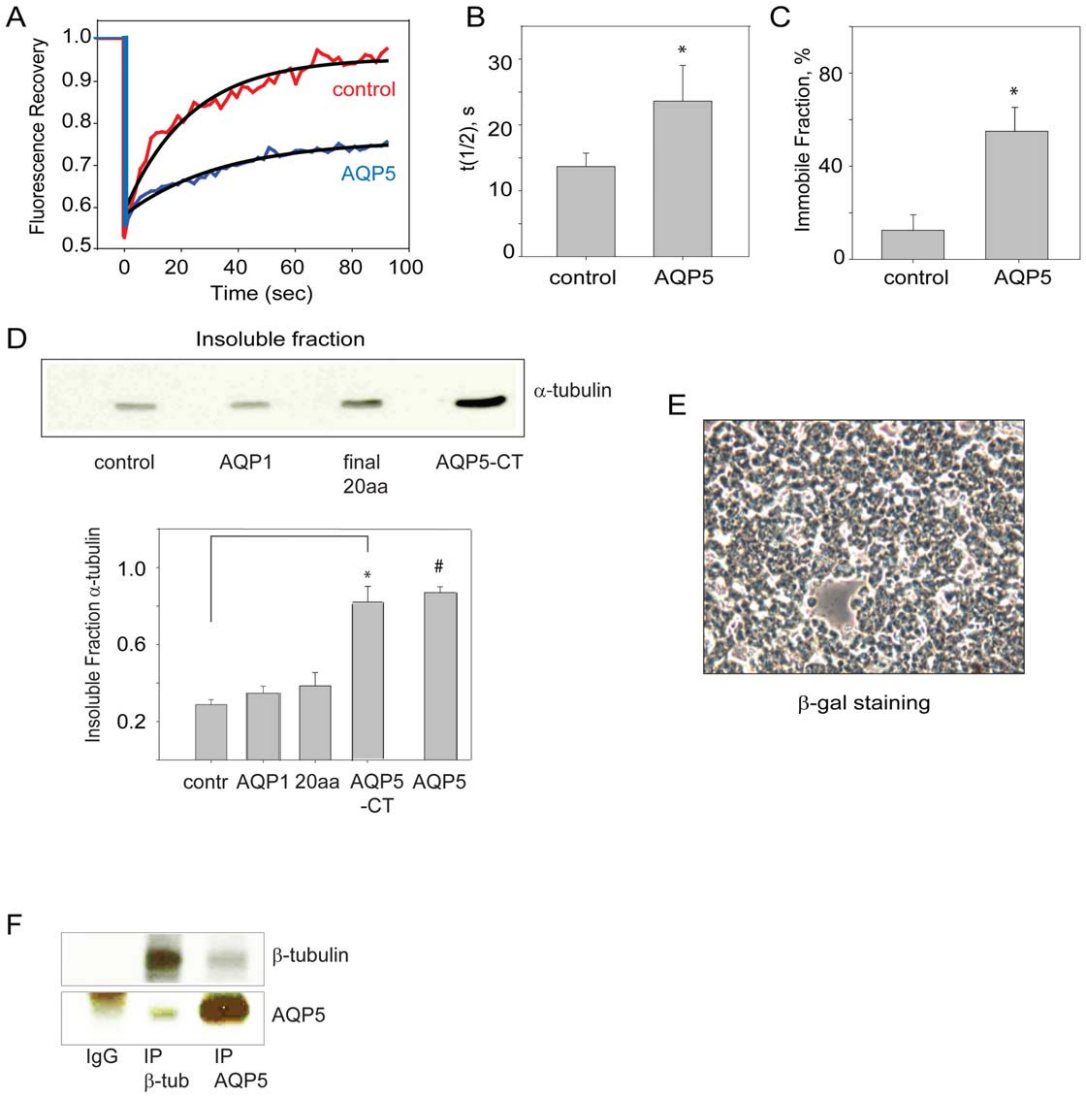
Overexpression of AQP5 led to delayed fluorescence recovery and increased MT stability. In primary human airway epithelial cells, AQP5 directly binds to tubulin. A. Using FRAP analysis of transduced 16HBE cells, which do not naturally express AQP5, we found that AQP5 delayed fluorescence recovery of GFP-α-tubulin after photobleaching. B. The measured recovery half-life (t_1/2_) of GFP-α-tubulin increased in cells expressing AQP5 compared to control cells (*, Student's t-Test (ST): *p*=0.01). C. AQP5-expressing cells also had a significantly increased immobile fraction (*, ST: *p*<0.01). D. AQP5-CT is sufficient to increase the insoluble fraction of tubulin in HEK cells. Control β-galactosidase, full-length AQP1, the final 20 amino acids of AQP5 or the entire AQP5-CT (carboxyl-terminal 40 amino acids) were transfected into HEK cells using the protein transfection reagent Chariot (Activemotif) and insoluble MT fractions were collected. Full-length AQP5 data was reproduced from Fig. 2 and shown here for direct comparison. Detection of the soluble fraction was minimal in these cells. E. β-galactosidase-staining in control cells confirmed that the transfection efficiency was 90–100%. E. NHBE cells, with immunoprecipitation of b-tubulin, there is pull-down of AQP5, and similarly with immunoprecipitation of AQP5, there is pull-down of b-tubulin. An IgG immunoprecipitation was performed as a control (n=4, per condition).

The steady state levels of MT assembly are established by a balance between assembly (nucleation and elongation) and disassembly. Our *in vitro* observations (above) indicate that AQP5 could either promote assembly and/or stabilize MTs, both of which would increase the insoluble fraction of tubulin (Fig. 1, 2, 3, 4). We transduced a human epithelial cell line which does not express AQP5 (16HBE cells), with either adeno-AQP5 or control adenovirus, and using fluorescence recovery after photobleaching (FRAP) we observed that cells expressing AQP5 had reduced fluorescence recovery, including increased half-life and immobile fraction (Fig. 5A–C). These data then indicate that AQP5 promotes MT stability in intact cells.

To determine if AQP5-CT could similarly stabilize MTs in cells, we transfected purified AQP5-CT protein into HEK cells. Direct transfection of purified protein was necessary as recombinant expression from an episomal plasmid did not yield peptide expression, presumably due to immediate degradation. After introduction of AQP5-CT, the tubulin insoluble fraction increased. This stabilization was specific to AQP5-CT as the shorter 20 a.a. tail region of AQP5 and AQP1 failed to increase the insoluble tubulin fraction (Fig. 5D). This indicates that a critical region in AQP5-CT lies in the first half of the final 40 amino acids of the protein. To confirm the transfection efficiency for these experiments, we followed β-galactosidase activity, confirming 90–100% transfection efficiency (Fig. 5E). In order to confirm that this stabilization is due to direct interation between AQP5 and MTs, we performed co-immunoprecipitation in NHBE cells. Immunoprecipitation of β-tubulin resulted in AQP5 pull-down. Similarly, immunoprecipitation of AQP5 resulted in pull-down of β-tubulin (Fig. 5F).

**Figure 6:**
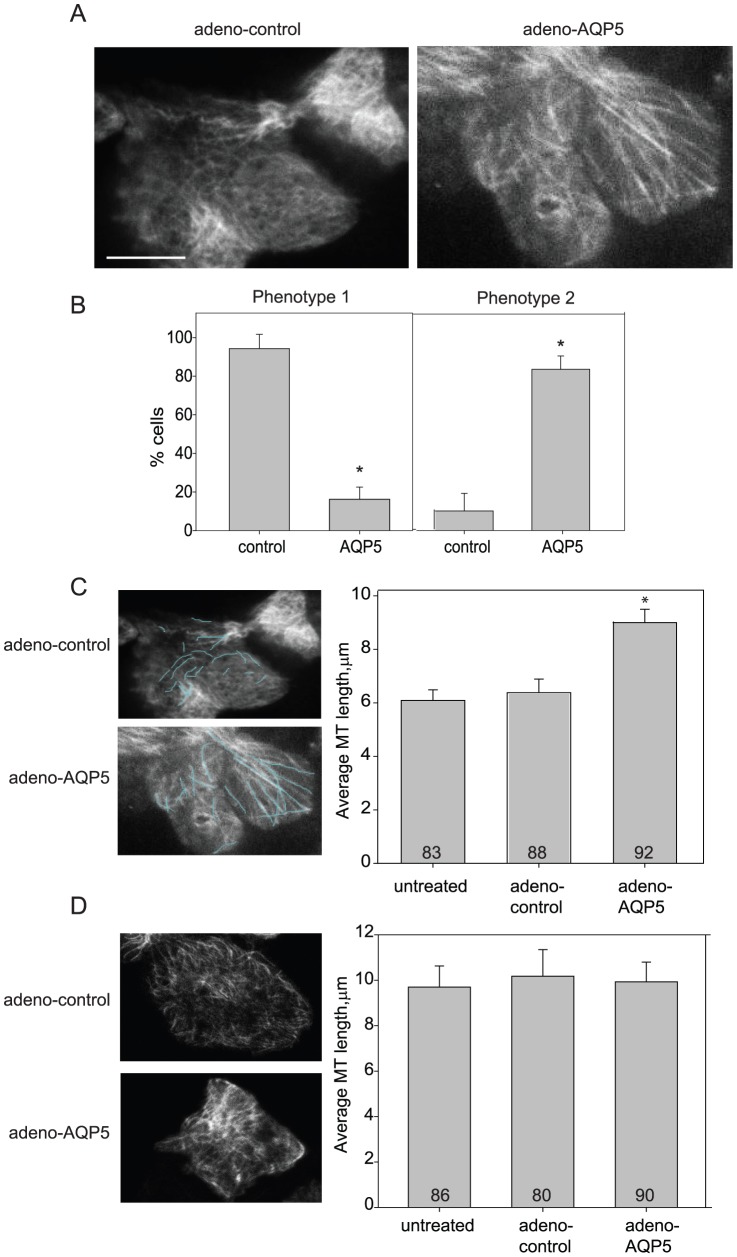
Total internal reflection fluorescence (TIRF) imaging showed that AQP5 altered MT structure at apical surfaces. A. Cells transduced with control adenoviruses (adeno-control) show a MT meshwork at their apical surfaces. Cells expressing AQP5 (adeno-AQP5) had longer, more aligned MTs at their apical surfaces. Scale bar, 10 µm. B. Cells expressing AQP5 were significantly more likely to have long and straight apical MTs. MT phenotypes were grouped into two categories, phenotype 1 (panel A, adeno control (n=22 cells) image is an example) and phenotype 2 (panel A, adeno-AQP5 (n=20 cells) image is an example). Cells within each treatment group were sorted based on image appearance. The frequency of cells that had either phenotype for each condition is plotted on the bar graphs. The two groups were significantly different (Comparison of Proportions (CP): z=4.6; *p*<0.0001). C. Cells expressing AQP5 had longer apical MTs than control cells had. Images show examples of individual traces MTs. Bar graph shows the average length per condition (ANOVA one-way: *p*=0.001). Using live TIRF, microtubules visualized on the apical surface were measured using ImageJ. 8–10 microtubules were measured per cell across 20–22 cells per treatment group. Total MTs analyzed is shown on the bar graph. D. Analysis of the basolateral surface showed no significant difference in MT length per condition. Bar graph shows the average length per condition with total number of MTs sampled depicted (8–10 cells per treatment group).

Lastly, to better observe the effect of AQP5 on MTs in cells, we performed total internal reflection fluorescence (TIRF) microscopy on 16HBE cells ±AQP5. TIRF imaging of the apical surface revealed a distinctive difference in the MT organization ± AQP5 (Fig. 6A). Without AQP5, the MTs were organized into a meshwork, where little overall pattern was visible. In contrast, in cells expressing AQP5, the MTs were much more highly organized, running parallel for greater distances, and had an overall greater end to end length visible in the TIRF field. The cells were grouped into one or the other category based on image appearance and the frequency of cells that had either phenotype was analyzed (Fig. 6B). In addition, cells expressing AQP5 had 50% longer apical MTs than control cells (Fig. 6C). In contrast, TIRF imaging of the basolateral membrane did not identify differences in microtubule length or structure (Fig. 6D).

## Discussion

Since their discovery in the early 1990's, the primary focus of aquaporin research has been on the transcellular transport of water and other small solutes across the cell membrane. Novel roles for aquaporins besides bulk transport of water have been identified. Saadoun et al [31] first proposed roles for AQP1 in cell migration and angiogenesis. Since then, other aquaporins have been identified to have similar roles in cell migration and proliferation [32], [33], [34], [35]. However, even these novel roles are thought to occur via local water flux driven by small osmotic changes due to actin polymerization-depolymerization and transmembrane ion fluxes [32]. Others have proposed that AQP0 could play a role in modulating cell-cell contacts, potentially via interaction with connexins [36], [37], [38], [39], [40]. While AQP4 has also been postulated to participate in mediating cell-cell contacts [41], this has been met with some controversy [42]. To our knowledge, in this report we provide the first description of a role for an aquaporin in directly associating with and modulating the cytoskeleton.

Twelve mammalian homologues have been identified, with distinct cellular and subcellular distributions [2]. It has been hypothesized that different homologues are required to achieve distinct regulation of water homeostasis in different cells and organs [2]. In addition, localization and regulation of each aquaporin is distinct, and therefore allows for further fine-tuning of water regulation in cells. However, our study demonstrates an additional rationale for multiple aquaporins. In addition to its role in water transport, AQP5 directly binds to MTs and increases their assembly. This function is at least relatively specific, since AQP1 did not alter MT dynamics. Our data indicates that AQP5 increases MT assembly primarily by stabilizing MTs, but in addition, our cell-free assay indicates that AQP5 also can promote MT nucleation. It is not known if this latter mechanism occurs *in vivo*.

Our data shows that the carboxyl-terminus– the longest intracellular portion of the protein– is sufficient to mediate this increase in MT assembly. The carboxyl-terminus is distinct from the conserved segment of the aquaporins, the Asn-Pro-Ala (NPA) motif, that are critical in regulating water transport through the pore and through the membrane [43]. When the carboxyl-terminus of AQP5 and AQP1 are aligned, only 11/35 (32%) amino acids are identical, so it is perhaps not surprising that that the two proteins do not function similarly when it comes to MT polymerization.

The fact that the carboxyl-terminus is sufficient to allow for MT assembly raises other interesting considerations. While aquaporins are present in the membrane as tetramers, the water channel forms through the center of the monomer, rather than the center of the tetramer, as is often observed in ion channels [44]. Structurally, based on analysis of AQP1, each monomer is positioned such that the outside face of the tetramer is hydrophobic, and the center of the tetramer is hydrophilic [43]. Further, in AQP5, a lipid occludes the putative central pore [44]. Given the tetrameric structure, one might speculate whether this organization allows for juxtaposition of four AQP5-CT domains, further enhancing AQP5 effects on MT stabilization.

Previous work from our lab and others has shown that AQP5 modulates paracellular permeability in epithelial cells [5], [6]. This is associated with changes in proteins mediating cell-cell contacts. However, the mechanisms by which changes in an apically expressed water channel alter proteins in the lateral membrane are not clear. Our study provides insight into one potential mechanism, by showing that AQP5 can directly modulate MT stability. Further molecular definition of how altered MT assembly affects adherens junction and desmosomal proteins awaits elucidation and these responses in cell-cell contacts maybe cell-type specific. Other microtubule associated proteins such as MAP4 [45] are present in airway epithelial cells, suggesting that MAP4, along with AQP5, could modulate airway epithelial microtubule dynamics. However, AQP5 is tightly regulated in lung epithelial cells, and dynamically responds to several physiologic and pathologic stimuli including TNFα [46], cAMP [47], [48], [49], osmotic stress [26], [50], [51], [52], [53], [54], LPS [55], [56] and shear stress [6]. While it has been hypothesized that tight regulation of AQP5 may be needed to control transmembrane water flux, however, coordination of MT dynamics with consequential changes in paracellular permeability is an alternate explanation for this level of regulation. AQP5 can be internalized in response to certain stimuli such as cAMP in as little as two minutes [48] and be degraded in response to osmotic stress in thirty minutes [50]. Clearly, AQP5 is subjected to multiple levels of regulation, leading to changes in paracellular permeability on different time-scales in response to different types of luminal stimuli. While our study indicates that AQP5 can directly mediate changes in microtubule dynamics, we do not rule out the possibility of subsequent indirect effects on microtubule polymerization also leading to the changes in MT stability. To our knowledge, this is the first demonstration that an aquaporin can directly mediate changes in cytoskeletal organization via a mechanism independent of water transport, providing yet another novel role for an aquaporin.

## Materials and Methods

### Materials

Unless specified, all reagents were purchased from Sigma.

### Cell culture, stimulation and immunoblotting

Primary human bronchial epithelial cells (NHBE) (Lonza) were grown on collagen-coated inserts (Falcon) at 37°C with 5% CO_2_ in specified media and maintained at an air-liquid interface for 6–9 weeks before study; transepithelial resistance (TEER) was always >400 ohms when cells were used. Cells were harvested and lysed in RIPA buffer [6]. Chemiluminescence reagents and horseradish peroxidase-coupled secondary antibodies were from Amersham (Arlington Heights, IL). Bicinchoninic acid (BCA) protein assay kit was from Pierce (Rockford, IL). Antibodies to the carboxyl-terminus of human AQP5 were generated by our laboratory [57]. When equivalent loading could not be performed by a protein loading control, Ponceau S staining of the membrane was performed.

Immortalized human bronchial epithelial cells [6], [58], [59], [60] (16HBE, gift of Gary Cutting, Johns Hopkins but from ATCC) were cultured on inserts and infected with either control, GFP- or AQP5-expressing adenovirus (University of Iowa) as described previously [6]. HEK cells (ATCC) were cultured for specified experiments using MEM media with or without 15% serum.

### Protein concentration determination

Protein concentrations were estimated by the Bradford assay using BSA as a standard.

### Transfection

HBE cells were grown in chamber slides to 50 to 60% confluence and transiently transfected (1 µg/well) with HA-AQP5 [48] or control plasmid using FuGENE 6 (1.5 µl; Roche) according to the manufacturer's recommendations. In other experiments cells were transfected with GFP-tubulin (gift of Geri Kreitzer, Cornell University) as described above. In Chariot protein transduction studies, HEK cells were used cultured in 6-well dishes, with tranfection at 90–100% confluence. In specified studies cells were cultured on inserts and infected with either control-, GFP- or AQP5-expressing adenovirus. In specified studies NHBE cells were transduced with adenovirus expressing either shRNA directed against AQP5 or a non-targeting control. 10^9^ infectious particles/ml were used at the lowest concentration required for protein knockdown. Apical media was removed and basal media changed, and cells were used 4–5 days later.

### Confocal imaging

Confocal laser microscopy (Leica SP5) was performed on cells grown on inserts using antibodies against tubulin (Sigma) with appropriate secondary antibodies (Alexa 488 or Alexa 555; Molecular Probes). For specified experiments to detect microtubules, cells were fixed with −20°C methanol. In images obtained pre- or post- soluble tubulin extraction, cells were either fixed directly with −20°C methanol or after soluble tubulin extraction was performed, as described below. Images were obtained without adjusting gain between conditions, and for semi-quantitative assessment of intensity as demonstrated, the maximum projection intensity was obtained using Leica software over the entire field for the various samples.

### Shear stress

Fluid flow to generate shear levels of 1.5–3 dynes/cm^2^ was applied as described [6]. Fluid flow rates of 0.5–1 ml/min provide a shear stress consistent with the magnitude experienced by airway epithelial cells *in vivo* by airflow. Calculations for this are described [6].

### Permeability assay

Paracellular permeability of cultured cells was assessed by measuring the passage of 4 kD FITC-dextran across the monolayer as described [6].

### Expression and purification of AQP5

Human AQP5 was purified as described [48]. Briefly, AQP5 was purified from protease-deficient (*pep4* 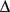) *Saccharomyces cerevisiae* expressing pYES2 (Invitrogen) containing human *AQP5*. Membranes were solubilized in 200 ml of buffer A with EDTA-free protease inhibitors (Roche Biochemicals)), loaded on a Ni-NTA agarose column (nickel-charged nitrilotriacetic acid, Qiagen), and eluted with 800 mM imidazole. This His-tag was cleaved with thrombin prior to testing.

### Microtubule–AQP5 co-sedimentation

Purified biotinylated tubulin was obtained from Cytoskeleton (Denver, CO), and *in vitro* sedimentation assays were performed per the manufacturer's instructions. AQP5 or an equal volume of AQP5 buffer as described above was added as indicated. MTs were pelleted at 100,000 g in a Beckman Ti-100 at 25°C. The resulting soluble and pellet proteins were separated by SDS-PAGE and silver staining performed to detect proteins.

### Microtubule polymerization assay

A tubulin polymerization kit was obtained from Cytoskeleton (Denver, CO) and used according to manufacturer's recommendations. Conditions were chosen to minimize polymerization of tubulin alone in order to detect an enhancer of tubulin polymerization. Therefore, no glycerol was added to the buffer. Polymerization is followed by fluorescence enhancement due to the incorporation of a fluorescent reporter into microtubules as polymerization occurs. The assay utilized 2 µM purified tubulin, which generates a polymerization curve, using absorbance readings at 340 nm to follow microtubule formation.

### Microtubule extraction protocol

Soluble and insoluble MT fractions were extracted as described [24]. Briefly, cells were rinsed with PBS, then 100 µl of PEM buffer containing 0.5% Triton X-100 and 25% glycerol was added for 45 seconds at 37°C to collect tubulin monomers (soluble tubulin). 150 µl of 2× RIPA buffer with a protease inhibitor cocktail was then added. Cell ghosts (polymerized tubulin) were lysed with 300 µl of 1× RIPA buffer and protease inhibitors. Samples were compared using immunoblotting as described above. Equivalent amounts of total protein as measured by BCA protein assay were loaded on the gels.

### Co-immunoprecipitation

The immunoprecipitation was performed as previously described [54]. After pre-clearing the samples with IgG and Protein A/G beads (Sigma), samples were incubated with an antibody against the protein of interest. The samples were analyzed by immunoblotting.

### Fluorescence Recovery after Photobleaching

16HBE cells were transfected with mCherry-tubulin as described above, and transduced with either adeno-AQP5, or adeno-control plasmid. Regions of interest (ROIs) located near the membrane within individual cells were photobleached and monitored for subsequent fluorescence recovery. The FRAP experiments were performed using a Nikon A1R Confocal Microscope equipped with the Perfect Focus module, an Akolab Microscope Stage Incubator, and a 60× oil-immersion objective. ROIs are defined (4.5 µm diameter circles) and two pre-bleaching frames were taken at 1.8% laser power for 3.9 s. Bleaching was achieved using 100% laser power at 561 nm for 15 frames for 7.6 s. 50 frames of post-bleach imaging were collected at 1.8% laser power for 1.37 minutes. The FRAP series was measured by using the offline version of NIS Element AR 3.10. The data measured are intensity vs. time for the ROIs. To determine recovery half-like (t ½), we determined the k constant using the equations: I=a*(1−exp(-kt))+c, where I is the measured intensity, using a best-fit analysis for k, a, and c. Then t ½ per condition was determined using ln (0.5/-k). The immobile fraction was calculated by the following equation: Immobile fraction=(1−f)/(1−g), where f=plateau level after recovery and g=level after initial photobleaching. Averages were obtained using 6–8 samples per condition. We corrected for photobleaching during the recovery phase by selecting a distinct ROI within the cells. In addition, background correction was performed by comparing to an ROI outside the cell.

### Total Internal Reflection Fluorescence

To assess the microtubule dynamics at the apical surface, TIRF movies were collected with a 2 s intervals using a 60× objective and 1.6× optovar. MDCK cells stably expressing GFP-tubulin were cultured on #1.5 8 mm round cover slips (Electron Microscopy Sciences 72296-08) for 48 hours before imaging, along with AQP5 or null adenovirus. Prior to imaging, the cover slips were reversed apically onto glass-bottom culture dishes (MatTek P35G-1.5-10-C). The inverted coverslips were also gently compressed using a thin sheet of agarose to allow for better TIRF imaging.

### Chariot protein transfection

Peptide introduction into cells was performed according to manufacturer's protocol (Activemotif). Briefly, a chariot-peptide complex was formed by incubation for 30 min. after which it was added to serum free cell media and the plate was gently rocked to ensure even delivery. Cells were incubated for 1.5–2 hrs, after which soluble and insoluble MT fraction were collected as described.

### Statistics

Statistical analysis was performed using STATA 9 (Stata Corporation). One-way ANOVA, Student's t-test (ST) and Comparison of Proportions (CP) were used as appropriate. The specific test is indicated along with *p*-values.
